# Analysis of the risk factors for intraoperative acute diffuse brain swelling in patients with isolated traumatic acute subdural haematomas

**DOI:** 10.1186/s12893-022-01637-5

**Published:** 2022-05-14

**Authors:** Ruhong Wu, Chunbo Liu, Tao Ma, Geng Jia, Huaping Qin

**Affiliations:** Department of Neurosurgery, The Third Affiliated Hospital of Soochow University, No.185, Juqian Road, Changzhou, 213003 China

**Keywords:** Acute subdural hematoma, Acute diffuse brain swelling, Decompressive craniectomy

## Abstract

**Background:**

The purpose of this retrospective study was to investigate the risk factors for intraoperative acute diffuse brain swelling in patients with isolated traumatic acute subdural haematomas (ASDH).

**Methods:**

A total of 256 patients who underwent decompressive craniectomy for isolated traumatic ASDH between April 2013 and December 2020 were included. We evaluated the risk factors for intraoperative acute diffuse brain swelling using a multivariate logistic regression analysis.

**Results:**

The incidence of intraoperative acute diffuse brain swelling in patients with isolated traumatic ASDH was 21.88% (56/256). Dilated pupils (OR = 24.78), subarachnoid haemorrhage (OR = 2.41), and the time from injury to surgery (OR = 0.32) were independent risk factors for intraoperative acute diffuse brain swelling, while no independent associations were observed between these risk factors and sex, age, the mechanism of injury, the Glasgow Coma Scale score, site of haematoma, thickness of haematoma, midline shift and the status of the basal cistern, although the mechanism of injury, the Glasgow Coma Scale score and the status of the basal cistern were correlated with the incidence of intraoperative acute diffuse brain swelling in the univariate analyses.

**Conclusions:**

This study identified the risk factors for intraoperative acute diffuse brain swelling in patients with isolated traumatic ASDH. An increased risk of intraoperative acute diffuse brain swelling occurs in patients with bilaterally dilated pupils, subarachnoid haemorrhage and a shorter time from injury to surgery. These findings should help neurosurgeons obtain information before surgery about intraoperative acute diffuse brain swelling in patients with isolated traumatic ASDH.

## Background

Acute subdural haematoma (ASDH) is a common and serious injury in traumatic brain injury (TBI) patients. Decompressive craniectomy (DC) is recommended when patients have a decreased level of consciousness, a greater size of the haematoma, a midline shift, or a basal cistern obliteration [1]. Acute encephalocele is a very dangerous and urgent situation during DC. The main causes of acute encephalocele include a contralateral subdural haematoma, a contralateral epidural haematoma and acute diffuse brain swelling [2, 3]. Acute diffuse brain swelling is one of the leading causes of intraoperative acute encephalocele, and it also has the highest mortality. Neurosurgeons can manage remote intracranial haematomas by contralateral craniotomy but are often struggle to manage diffuse brain swelling due to a lack of an effective treatment method. Acute diffuse brain swelling during DC is often accompanied by an acute drop in blood pressure, and the mortality is very high. This situation is a significant concern for all neurosurgeons. However, few reports have evaluated the risk factors for intraoperative acute diffuse brain swelling in patients with isolated traumatic ASDH. This information is urgently needed in clinical work to estimate the incidence of intraoperative acute diffuse brain swelling based on preoperative clinical and imaging data, and this information can help neurosurgeons accurately determine the surgical risk and to provide reasonable suggestions to patients’ families. In this study, we aimed to explore the risk factors for intraoperative acute diffuse brain swelling in patients with isolated traumatic ASDH.

## Methods

### Patient population

This retrospective study was performed on 256 patients who underwent surgery after being diagnosed with isolated traumatic ASDH at our neurosurgical department between April 2013 and December 2020. We set the inclusion and exclusion criteria for this retrospective study, with the final number of cases decided according to the inclusion and exclusion criteria. The inclusion criteria for this retrospective study were as follows: (1) ASDH caused by trauma, (2) ASDH located on the supratentorial region of the brain, (3) emergency DC performed immediately after admission, and (4) age between 18 and 80 years old. The following exclusion criteria were applied: (1) patients with penetrating head injuries, acute epidural haematomas, ASDH of the posterior fossa, intracerebral haematomas and serious extracranial injuries; (2) patients whose time from injury to surgery exceeded 8 h; (3) patients whose preoperative blood pressure was lower than 90/60 mmHg; (4) patients who took anticoagulants or antiplatelet drugs; and (5) patients who had severe diseases of the heart, lungs, liver, kidneys, or haematologic system. Age, sex, the mechanism of injury, the Glasgow Coma Scale score, pupil reaction, site of haematoma, thickness of haematoma, midline shift, subarachnoid haemorrhage (SAH), status of the basal cistern and time from trauma to surgery were investigated. The study was approved by the Ethics Committee of the Third Affiliated Hospital of Soochow University.

### General patient management

According to the above inclusion and exclusion criteria, we finally selected and analysed 256 patients. We categorized all of the variables into three groups according to which variables might be related to the risks of intraoperative acute diffuse brain swelling of isolated traumatic ASDH, as follows: (1) clinical variables including sex, age, the mechanism of injury, the Glasgow Coma Scale (GCS) score, and pupillary reaction; (2) computerized tomography (CT) variables included the site of haematoma, midline shift, thickness of the haematoma, the status of the basal cistern, and SAH; and (3) surgical variable was the time from trauma to surgery.

After an isolated traumatic ASDH was diagnosed and when there were indications for surgery, each patient underwent DC with a question mark incision. First, a 5 cm incision was made in the temporal area along the question mark line, and a hole was drilled into the skull. Then a small hole was cut in the dura to try to slowly release the liquid ASDH, and then a DC was performed. The size of the skull bone flap was approximately 10 cm by 10 cm. The dura was opened, and the haematoma was evacuated in all of the patients. The criteria for intraoperative acute diffuse brain swelling were as follows: after opening the dura, an encephalocele immediately appeared, and brain tissue protruded from the inner edge of the bone window ≥ 1 cm; the degree of encephalocele progressively worsened; the texture of the brain tissue was hard; and there was almost no obvious brain pulsation. At the same time, acute encephalocele may be accompanied by a progressive decrease in the patient’s blood pressure. Blood transfusions and volume expansion could not correct the patient’s hypotension. After emergency closing of the cranial cavity, a brain CT scan was immediately performed and did not find new haematomas.

### Clinical variables

The mechanism of injury included motor vehicle accidents, falls, and falls from a height. The GCS scores of the patients were determined on admission, and all patients were divided into three categories (3–5, 6–8, 9–15). The preoperative pupillary reaction was divided into two categories: no dilated pupils and unilaterally dilated or bilaterally dilated pupils.

### CT variables

**Fig. 1:**
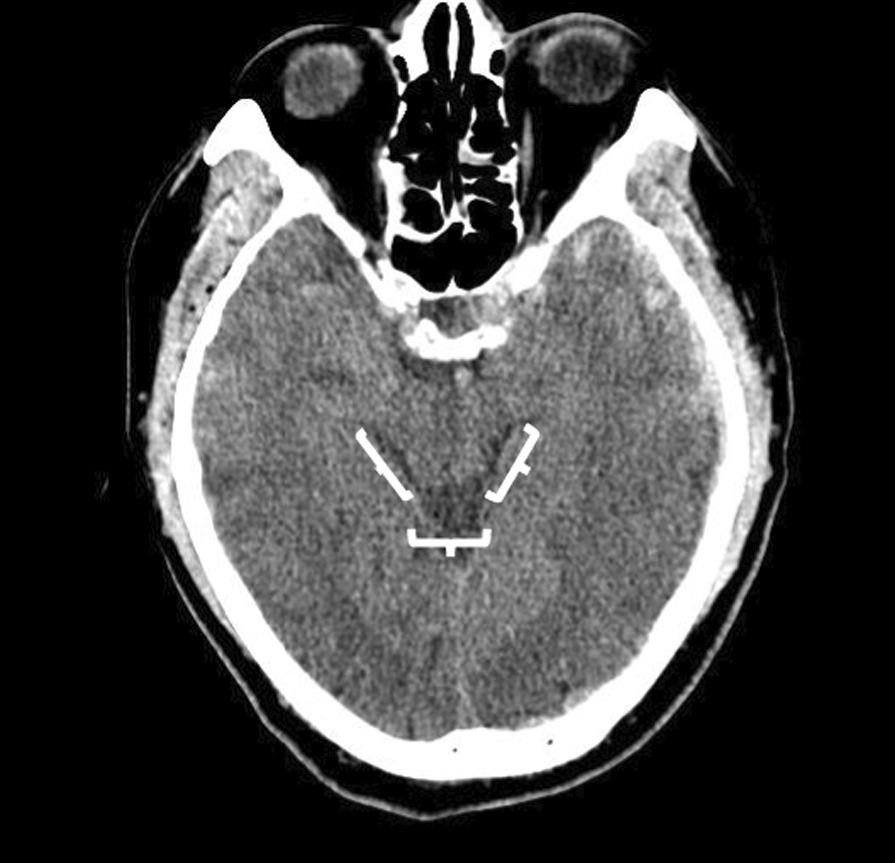
Evaluation of the basal cisterns on computed tomographic scan

The maximum thickness (< 5 mm, 5–9.9 mm, 10–14.9 mm, ≥ 15 mm) and midline shift at the septum pellucidum (< 5 mm, 5–9.9 mm, 10–14.9 mm, ≥ 15 mm) were analysed according to preoperative CT scans. The condition of the basal cistern was evaluated using the axial image at the level of the midbrain, which was then divided into three branches (one posterior and two laterally) (Fig. 1). Each branch was assessed separately to determine whether it was open or occluded. For the logistic regression analysis, patients were divided into four categories (0, 1, 2, 3 branches occluded).

### Surgical variables

The American College of Surgeons Committee on Trauma set a demarcation line for whether patients with traumatic ASDH undergo surgery within 4 h [4]. According to the demarcation line, we divided all patients into two categories (< 4 h, ≥ 4 h) according to the time from trauma to surgery.

### Statistical analysis

Statistical analyses of the data were carried out using SPSS 22.0 software IBM Corp., Armonk, New York, USA). Discrete variables were compared using the Chi-square test, and continuous variables were compared using the Mann–Whitney U test. All variables were used as candidates and put into a multivariate logistic regression model to identify which variables were independently associated with the risk factors for intraoperative acute diffuse brain swelling in patients with isolated traumatic ASDH, and all variables with p < 0.05 were considered to have statistical significance.

## Results

### Univariate analysis of the risk factors for intraoperative acute diffuse brain swelling

Of the 256 patients, 56 patients experienced intraoperative acute diffuse brain swelling, and the overall incidence was 21.8%. The median age of these patients was 57 years (range 18–79 years). Male patients constituted 72.3% of the study population, which included 185 men and 71 women. Eleven variables contributing to intraoperative acute diffuse brain swelling were analysed separately in the univariate analyses. Consequently, six factors were found to be significantly related to intraoperative acute diffuse brain swelling (Table 1).

**Table 1:** Variables related to the risk factors for intraoperative acute diffuse brain swelling in patients with isolated traumatic ASDH (n = 256)

Variables	No acute diffuse brain swelling	Acute diffuse brain swelling	Statistical values	*p* value
Totals	200	56 (21.8%)		
Sex			0.731	0.393
Male	142	43 (23.2%)		
Female	58	13 (18.3%)		
Age (years)	57.5	55.5	5399	0.681*
Mechanism of injury			8.432	0.015
Motor vehicle accident	121	43 (26.2%)		
Fall down	55	5 (8.3%)		
Falls from a height	24	8 (25%)		
GCS			35.198	< 0.001
3–5	121	55 (31.3%)		
6–8	64	1 (1.5%)		
9–15	15	0 (0%)		
Dilated pupils			52.332	< 0.001
Absent or unilateral	120	3 (2.4%)		
Bilateral	80	53 (39.8%)		
Site of haematoma			0.333	0.56
Left	98	25 (20.3%)		
Right	102	31 (23.3%)		
Thickness of haematoma (mm)			4.303	0.231
< 5	10	4 (28.6%)		
5–9.9	87	16 (15.5%)		
10–14.9	72	26 (26.5%)		
≥ 15	31	10 (24.4%)		
Midline shift (mm)			2.170	0.538
< 5	8	4 (33.3%)		
5–9.9	52	13 (20.0%)		
10–14.9	89	21 (19.1%)		
≥ 15	51	18 (26.1)		
SAH			9.131	0.003
No	117	20 (14.6%)		
Yes	83	36 (30.3%)		
Condition of the basal cistern			38.450	< 0.001
0	9	0 (0.0%)		
1	30	0 (0.0%)		
2	83	9 (9.8%)		
3	78	47 (37.6%)		
The time from injury to surgery (h)			11.979	0.001
< 4	122	48 (28.2%)		
≥ 4	78	8 (9.3%)		

*GCS* Glasgow Coma Scale, *SAH* subarachnoid haemorrhage

^*^Mann–Whitney U test

The incidence of intraoperative acute diffuse brain swelling had no significant correlation with sex or age (*p* > 0.05). The incidence of intraoperative acute diffuse brain swelling was found to have a significant correlation with the mechanism of injury (*p* < 0.05). Of the 164 patients with motor vehicle accidents, 43 (26.2%) experienced intraoperative acute diffuse brain swelling. In contrast, only 5 (8.3%) of the 60 patients who suffered falls experienced intraoperative acute diffuse brain swelling. The incidence of intraoperative acute diffuse brain swelling showed a significant correlation with the GCS score (*p* < 0.05). Of the 176 patients with GCS scores ranging from 3 to 5, 55 (31.1%) experienced intraoperative acute diffuse brain swelling. In contrast, only 1 (1.5%) of the 65 patients with GCS scores ranging from 6 to 8 experienced intraoperative acute diffuse brain swelling, and no patients with GCS scores ranging from 9 to 15 experienced intraoperative acute diffuse brain swelling. The incidence of intraoperative acute diffuse brain swelling was found to have a significant correlation with dilated pupils (*p* < 0.05). Of the 133 patients with bilaterally dilated pupils, 53 (39.8%) experienced intraoperative acute diffuse brain swelling. In contrast, intraoperative acute diffuse brain swelling occurred in only 3 (2.4%) of the 123 patients, with no dilated pupils or unilateral pupil dilation.

The incidence of intraoperative acute diffuse brain swelling had no significant correlation with the site of haematoma, the thickness of haematoma or midline shift (*p* > 0.05). The incidence of intraoperative acute diffuse brain swelling showed a significant correlation with SAH (*p* < 0.05). Of the 119 patients with SAH, 36 (30.3%) experienced intraoperative acute diffuse brain swelling. In contrast, acute diffuse brain swelling occurred in only 24 (14.6%) of the 137 patients without traumatic SAH.

The condition of the basal cistern was significantly correlated with the incidence of intraoperative acute diffuse brain swelling (*p* < 0.05). The incidence (37.6%) of intraoperative acute diffuse brain swelling in patients with three occluded branches was significantly higher than that in the other three groups. The time from injury to surgery significantly influenced the incidence of intraoperative acute diffuse brain swelling (*p* < 0.05). The incidence of intraoperative acute diffuse brain swelling (28.2%) in patients undergoing surgery within 4 h after injury was higher than that in the other patients (*p* = 0.001).

### Multivariate analysis of risk factors for acute diffuse brain swelling

The multivariate logistic regression analysis showed that dilated pupils, SAH, and the time from injury to surgery were independent predictors of the risk factors for intraoperative acute diffuse brain swelling in patients with isolated traumatic ASDH. The odds ratios (ORs) of these three variables and the regression formulas are summarized in Table 2. No independent association was observed between the incidence of intraoperative acute diffuse brain swelling and age, sex, the mechanism of injury, the GCS score, the site of haematoma, the thickness of haematoma, midline shift or the condition of the basal cistern.

**Table 2:** Independent predictors of the risk factors for intraoperative acute diffuse brain swelling in patients with isolated traumatic ASDH by multivariate logistic analysis

Factors	OR (95% CI)	*p* value
Dilated pupils		
Absent or unilateral	1.00	
Bilateral	24.78 (7.39–83.09)	0.000
SAH		
No	1.00	
Yes	2.40 (1.19–4.84)	0.014
The time from injury to surgery		
< 4	1.00	
≥ 4	0.32 (0.13–0.77)	0.011

*SAH* subarachnoid haemorrhage, *CI* confidence interval, *OR* odds ratio

## Discussion

Traumatic ASDH is the most common condition in severe head trauma patients. It occurs in approximately 30% of patients with severe head injury and has reported mortality rates up to 60% for patients treated within the last decade [5–7]. This leads to serious public health, social, and economic concerns. For traumatic ASDH with surgical indications, DC is the most commonly used surgical technique [8].

Previous reports have shown that an acute encephalocele significantly increase the mortality of patients with acute ASDH [9]. Almost every neurosurgeon will experience it. The common causes include contralateral subdural or epidural haematomas, epidural haematomas in the confluence of sinus areas, acute traumatic cerebral infarctions, and acute diffuse brain swelling [10]. In these cases, acute diffuse brain swelling is particularly dangerous, with a high mortality and a poor prognosis [9]. Judging the risk of intraoperative acute diffuse brain swelling in patients with traumatic ASDH based on preoperative clinical data and imaging data is a problem that our neurosurgeon is more concerned about because this information can help us more accurately determine the operation risks and give the patients’ families more accurate advice.

Acute diffuse brain swelling usually occurs rapidly after the dura is opened, which is different from acute epidural haematomas or acute subdural haematomas in remote sites because these usually occur after a period of time after the dura is opened. The cerebral vein is in a state of obvious congestion with dark purple colouring, and there may be accompanying cerebral venous rupture and haemorrhage. The texture of the brain tissue becomes hard without brain pulsation, and brain tissue “fermentation”-like bulges are usually accompanied by a sharp drop in blood pressure, while blood transfusions or fluid rehydration often cannot maintain the patient’s blood pressure. The prognosis is very poor, with most patients dying within 7 days after the operation. The main mechanism of intraoperative acute diffuse brain swelling is that an external force causes damage to the cerebrovascular motor centre after brain injury, which in turn causes a loss of cerebrovascular autoregulation. Then, the brain tissue becomes congested, and the cerebral blood flow and blood volume increase rapidly [11], which leads to acute diffuse brain swelling. When the dura is opened during DC, the intracranial pressure (ICP) drops rapidly, and the cerebral blood vessels become acutely dilated. During this time, the brain tissue is in a hyperperfusion condition, and the cerebral vascular tension centre is damaged [12, 13], which makes it difficult to retract the blood vessels. Then, diffuse brain swelling occurs.

We observed that the incidence of intraoperative acute diffuse brain swelling was 21.8% in 256 surgical patients with isolated traumatic ASDH. The multivariate logistic regression analysis indicated that dilated pupils, SAH, and the time from injury to surgery were independently associated with the incidence of acute diffuse brain swelling.

In this study, the incidence of intraoperative acute diffuse brain swelling in patients who were injured in motor vehicle accidents was higher than that in patients injured from falls, and this difference was statistically significant. This may indicate that the brain injury sustained in a car accident is more complicated because it is a more violent event, which easily causes rotational movement of the head, and the shearing force generated by this rotation leads to extensive and serious damage to the brain and vascular motor centres, expanding the cerebral blood vessels and causing diffuse brain swelling.

Among the 256 surgical patients with traumatic ASDH, the incidence of intraoperative acute diffuse brain swelling in patients with bilaterally dilated pupils was significantly higher than that in patients without dilated pupils and patients with unilateral dilated pupils. TBI can cause rupture of the cerebral arteries and veins and swelling of the cerebral hemispheres. Due to the space-occupying effect of the subdural haematoma and the swollen brain tissue, the ICP is significantly increased, resulting in obstruction of venous return and cerebral oedema, which in turn increase the ICP, causing brain herniation. Cerebral herniation leads to a severe cerebral venous return disorder, which forms a vicious cycle. This is closely related to the theory of the pathogenesis of intraoperative acute diffuse brain swelling [13, 14]. Dilated pupils in patients with TBI not only indicate that the patient has herniated or is in the preherniation stage but also indicate that the prognosis is poor. According to some scholars, the mortality of patients with bilateral dilated pupils can reach as high as 64–88.1% [15, 16]. Bilaterally dilated pupils indicate that the patient’s brainstem has been more severely compressed, the patient’s brain swelling is more serious, and these findings are more likely to cause intraoperative acute diffuse brain swelling.

Although the multivariate logistic regression analysis indicated that the GCS score was not independently associated with the incidence, the univariate analysis indicated that the GCS score was highly correlated with the incidence of acute diffuse brain swelling during surgery. Patients with lower GCS scores had a higher incidence of intraoperative acute diffuse brain swelling than patients with higher GCS scores. A GCS score lower than 5 illustrates that the patient’s primary brain injury is severe, and the shear stress generated by rotating external force damages the vascular motor centres, resulting in the loss of cerebrovascular autoregulation and acute cerebrovascular expansion. The increase in the cerebral blood volume leads to brain swelling, which can easily lead to intraoperative acute diffuse brain swelling. Patients with dilated pupils tend to have lower GCS scores than the patients with nondilated pupils. It is recognized that the state of the patient’s pupil is closely related to the prognosis. According to previous reports, mortality is 100% in patients with a GCS score of 3 points combined with bilateral pupil dilation [17]. Analysis of the prognostic factors in ASDH patients found that the GCS score is positively correlated with the prognosis of patients with ASDH, but it has not been proven to reflect the level of brainstem injury [18, 19]. Notably, evaluation of the GCS score is usually hindered by the presence of long-lasting sedatives and paralytics. Thus, the evolution of clinical status in patients who are paralyzed pharmacologically with long-acting agents cannot be determined. Therefore, the GCS score of the patient at admission may be lower than the patient’s true GCS score.

The multivariate logistic regression analysis showed that SAH was an independent risk factor for intraoperative acute diffuse brain swelling in patients with traumatic ASDH. Blood cells in the subarachnoid space can release substances that cause vasospasm, which can cause spasm of the cerebral arteries and can cause intracranial hypertension, cerebral oedema and even cerebral herniation [20], which may be why patients with SAH are prone to intraoperative acute diffuse brain swelling. The presence of SAH has been shown to be a strong predictor of both outcome and mortality in TBI patients [16, 21–23].

The multivariate logistic regression analysis indicated that the condition of the basal cistern was not independently associated with the incidence of intraoperative acute diffuse brain swelling, but the univariate analysis indicated that the incidence of intraoperative acute diffuse brain swelling in patients with three branches occluded was significantly higher than the incidence in the other three groups. The degree of compression of the basal cistern reflects not only the degree of ICP but also the degree of brainstem compression. The occluded basal cistern indicates that the supratentorial brain tissue compresses the brainstem, which is an important prognostic factor that is associated with poor outcome and death. The occluded basal cistern is often accompanied by local haemorrhage, which can further damage the brainstem. Studies have shown that patients with basal cistern occlusion, especially those with a significant occlusion, will have significantly lower GCS scores, indicating that their brainstems have primary or secondary damage; thus, these patients are prone to brain herniation [5]. Previous research found that patients with absent or compressed basal cisterns and a midline shift larger than 5 mm had the highest mortality rate of 44% [16]. Therefore, patients with severe basal cistern occlusion are more likely to experience intraoperative acute diffuse brain swelling.

At present, the impact of the timing of surgery on mortality and functional survival is still controversial. Most previous studies believe that prolonging the time to surgery may cause the best rescue window to be missed, resulting in a poor prognosis [5, 18, 24, 25], but other clinical scholars have found that performing surgery within 4 h versus 6 h after injury will not significantly impact mortality; however, with the extension of the timing of surgery, there is an increased risk of mortality and a decline in the functional recovery rate [26]. Here, the multivariate logistic regression analysis showed that the time from injury to surgery was an independent risk factor for intraoperative acute diffuse brain swelling. A time from injury to surgery longer than 4 h was a protective factor against intraoperative acute diffuse brain swelling, suggesting that prolonging the time from injury to surgery can help reduce the risk of intraoperative acute diffuse brain swelling. Kyu-Hong Kim reported that patients who underwent DC within 4 h had worse outcomes than those patients who underwent surgery at a later time point [27]. However, this does not suggest that delaying surgery leads to better outcomes. In our retrospective analysis, the high probability of acute diffuse brain swelling in patients undergoing surgery within 4 h may be because those patients who seem to be more severely ill and have a lower GCS score will be sent to the advanced neurosurgery trauma centre faster for emergency operative treatment. This selection bias will certainly skew the results, and this needs to be verified by more research. In addition, autoregulation of the cerebrovasculature was lost in the patients with bilaterally dilated pupils. Even if we performed a control gradient decompression during DC, the acute dilation of the cerebrovasculature still cannot be controlled, which ultimately causes intraoperative acute diffuse brain swelling. However, for patients with indications for emergency surgery, there is no doubt that the operations will be performed as soon as possible.

## Conclusions

This study identified the risk factors for intraoperative acute diffuse brain swelling in patients with isolated traumatic ASDH. An increased risk of intraoperative acute diffuse brain swelling was found in patients with bilaterally dilated pupils, SAH and a short time window between injury and surgery. The findings should help neurosurgeons obtain information before surgery about intraoperative acute diffuse brain swelling in patients with isolated traumatic ASDH. Some limitations of this study should be acknowledged that the retrospective design may lead to bias and uncontrollable confounding factors. First, in this study, early surgery can increase the probability of intraoperative acute diffuse brain swelling, the finding that a longer time to surgery could serve as a protective factor against acute diffuse brain swelling was contrary to common sense. Second, this retrospective study collected data from patients between the ages of 18 and 80. The main reason for this age range is that there are relatively few cases of ASDH in our medical centre among patients under the age of 18 or over the age of 80 who undergo emergency surgery. With the ageing of the population, future studies need to expand the age range of patients to determine whether elderly patients are prone to acute diffuse brain swelling. Third, early hypoxia or intubation time may also be factors in patients with acute diffuse brain swelling, but this retrospective study lacks detailed records of prehospital emergency care, including the time point at which some patients were intubated and whether they experienced aspiration or hypoxia, which may bias the results of the study. Fourth, not all patients underwent ICP monitoring. These limitations of this study need to be addressed through more research in the future.

## Data Availability

All patient data and clinical approaches adopted are contained in the medical files of The Third Affiliated Hospital of Soochow University. The bibliographic data of reference are available on PubMed and the conclusions are based on the opinion of the expert involved in this case. The data supporting the conclusions of this article are included within the article and its figures.

## Abbreviations

ASDH: Acute subdural hematoma
CT: Computed tomography
DC: Decompressive craniotomy
GCS: Glasgow Coma Scale
ICP: The intracranial pressure
SAH: Subarachnoid haemorrhage
TBI: Traumatic brain injury
